# Performance validity testing: the need for digital technology and where to go from here

**DOI:** 10.3389/fpsyg.2024.1452462

**Published:** 2024-08-13

**Authors:** John-Christopher A. Finley

**Affiliations:** Department of Psychiatry and Behavioral Sciences, Northwestern University Feinberg School of Medicine, Chicago, IL, United States

**Keywords:** performance validity, malinger, feign, digital, artificial intelligence, technology, neuropsychology, computerized

## Introduction

Neuropsychological testing can inform practitioners and scientists about brain-behavior relationships that guide diagnostic classification and treatment planning (Donders, 2020). However, not all examinees remain engaged throughout testing and some may exaggerate or feign impairment, rendering their performance non-credible and uninterpretable (Roor et al., 2024). It is therefore important to regularly assess the validity of data obtained during a neuropsychological evaluation (Sweet et al., 2021). However, performance validity assessment (PVA) is a complex process. Practitioners must know when and how to use multiple performance validity tests (PVTs) while accounting for various contextual, diagnostic, and intrapersonal factors (Lippa, 2018). Furthermore, inaccurate PVA can lead to erroneous and potentially harmful judgments regarding an examinee's mental health and neuropsychological status. Although the methods used to address these complexities in PVA are evolving (Bianchini et al., 2001; Boone, 2021), improvement is still needed.

Modern digital technologies have the potential to significantly improve PVA, but such technologies have not received much attention. Most PVTs used today are pencil-and-paper tests developed several decades ago (Martin et al., 2015), and digital innovations have largely been confined to computerized validity testing (see Table 1). Meanwhile, other areas of digital neuropsychology have rapidly expanded. Technologies can now capture high-dimensional data conducive to precision medicine (Parsons and Duffield, 2020; Harris et al., 2024), and this surge in digital assessment may soon become the rule rather than exception for neuropsychology (Bilder and Reise, 2019; Germine et al., 2019). If PVA does not keep pace with other digital innovations in neuropsychology, many validity tests and methods may lose relevance.

**Table 1:** Existing digital performance validity tests and methods.

**Material-specificity**	**Performance validity test/method**	**References**
Memory-focused freestanding PVTs	Memory integrated language test (MIL)	Finley et al., 2024b; Leese et al., 2024b
	Coin in hand–extended version	Daugherty et al., 2021
	Inventory of problems – memory (IOP-M)	Giromini et al., 2020; Erdodi et al., 2024
	DETECTS	Paulo and Albuquerque, 2019
	Computerized forced-choice test (CFCT)	Gutiérrez and Gur, 2011
	Medical symptom validity test (MSVT)	Green, 2004
	Word memory test (WMT)	Green, 2003
	Computerized test of memory malingering (TOMM)	Rees et al., 1998
	Computerized assessment of response bias (CARB)	Allen et al., 1997
	Tests of neuropsychological malingering (TNM)	Pritchard and Moses, 1992
Non-memory-focused freestanding PVTs	Making change test (MCT)	Finley et al., 2024b; Leese et al., 2024a
	The shell game task^*^	Bryant et al., 2023
	Multi-level pattern memory test (MPMT)	Omer and Braw, 2021
	Tests of attentional distraction (TOAD)	Morey, 2019
	Nonverbal medical symptom validity test (NV-MSVT)	Green, 2008
	Portland digit recognition test-computerized	Rose et al., 1995
	Victoria symptom validity test (VSVT)	Slick et al., 1995
	Forced choice test of nonverbal ability (FCTNV)	Frederick and Foster, 1991
	Multi-digit memory test (MDMT)	Bolter and Niccolls, 1991
Mixed freestanding PVTs	Pediatric performance validity test suite (PdPVTS)	McCaffrey et al., 2020
	Memory validity profile (MVP)	Brooks and Sherman, 2019; Brooks et al., 2019
Embedded PVTs/methods	Penn computerized neurocognitive battery (PennCNB)	Scott et al., 2023
	National Institutes of Health Toolbox^®^ (NIHTB)	Abeare et al., 2021
	MOXO-d-continuous performance test (CPT)	Berger et al., 2021; Winter and Braw, 2022
	Conners continuous performance test (CPT; Versions 2 and 3)	Ord et al., 2010; Erdodi et al., 2014; Shura et al., 2016; Sharland et al., 2018; Lichtenstein et al., 2019; Scimeca et al., 2021; Finley et al., 2023a,b; Robinson et al., 2023;
	Test of variables of attention (TOVA)	Leark et al., 2002; Marshall et al., 2010; Nicholls et al., 2020
	Automated neuropsychological assessment metrics (ANAM) performance validity index	Roebuck-Spencer et al., 2013; Meyers et al., 2022
	Immediate post-concussion assessment and cognitive testing (ImPACT)	Erdal, 2012; Schatz and Glatts, 2013; Lovell, 2015; Siedlik et al., 2015; Gaudet and Weyandt, 2017; Higgins et al., 2017; Manderino and Gunstad, 2018; Raab et al., 2020
	CNS vital signs battery	Brooks et al., 2014
	NeuroTrax battery	Hegedish et al., 2012; Bar-Hen et al., 2015

^*^Presented as a professional conference poster, not a published article.

This paper aims to increase awareness of how digital technologies can improve PVA so that researchers within neuropsychology and relevant organizations have a clinically and scientifically meaningful basis for transitioning to digital platforms. Herein, I describe five ways in which digital technologies can improve PVA: (1) generating more informative data, (2) leveraging advanced analytics, (3) facilitating scalable and sustainable research, (4) increasing accessibility, and (5) enhancing efficiencies.

## Generating more informative data

Generating a greater volume, variety, and velocity of data core and ancillary to validity testing may improve the detection of non-credible performance. With these data, scientists and practitioners can better understand the dimensionality of performance validity and assess it effectively, especially in cases without clear evidence of fabrication. However, capturing sundry data in PVA is challenging, as practitioners are often limited to a few PVTs throughout an evaluation that is completed in a single snapshot of time (Martin et al., 2015). Furthermore, many PVTs index redundant information because they have similar detection paradigms that generate only one summary cut-score (Boone, 2021). Digital technologies can address these issues by capturing additional aspects of performance validity without increasing time or effort.

Digitally recording the testing process is one way to generate more diverse data points than a summary score. Some process-based metrics are already employed in PVA, including recording response consistency and exaggeration across test items (Schroeder et al., 2012; Finley et al., 2024a). For example, Leese et al. (2024a) found that using a digital software to assess discrepancies between item responses and correct answers improved the detection of non-credible performance. Using digital tools to objectively and unobtrusively record response latencies and reaction times during testing is another useful process-based approach (Erdodi and Lichtenstein, 2021; Rhoads et al., 2021). Examinees typically cannot maintain consistent rates of slowed response latencies across items when attempting to feign impairment (Gutiérrez and Gur, 2011). Various software can record these process-based scores (e.g., item-level indices of response time, reliable span, and exaggeration magnitude) in most existing tests if they are migrated to tablets/computers (Kush et al., 2012). Recording both the process and outcome (summary scores) of test completion can index dimensions of performance validity across and within tests.

Technologies can also record biometric data ancillary to validity testing. Biometrics including oculomotor, cardiovascular, body gesture, and electrodermal responses are indicators of cognitive load and are associated with deception (Ayres et al., 2021). Deception is believed to increase cognitive load because it requires more complex processing to falsify a response (Dinges et al., 2024). Although deception is different from non-credible performance, neuroimaging research suggests non-credible performance can be indicative of greater cognitive effort (Allen et al., 2007). For this reason, technologies like eye-tracking have been used to augment PVA (Braw et al., 2024). These studies are promising, but other avenues within this literature have yet to be explored due to technological limitations. Fortunately, many technologies now possess built-in cameras, accelerometers, gyroscopes, and sensors that “see,” “hear,” and “feel” at a basic level, and may be embedded within existing PVTs to record biometrics.

Technologies under development for cognitive testing may also provide informative data that has not yet been linked to PVA. For example, speech analysis software for verbal fluency tasks (Holmlund et al., 2019) could identify non-credible word choice or grammatical errors. Similarly, digital phenotyping technologies may identify novel and useful indices during validity testing, such as keystroke dynamics (e.g., slowed/inconsistent typing; Chen et al., 2022) embedded with PVTs requiring typed responses. These are among many burgeoning technologies that can generate higher dimensional data needed for robust PVA without adding time or labor. However, access to a greater range and depth of data requires advanced methods to effectively and efficiently analyze the data.

## Leveraging advanced analytics

Fortunately, technologies can leverage advanced analytics to rapidly and accurately analyze a large influx of digital data in real time. Although several statistical approaches are described within the PVA literature (Boone, 2021; Jewsbury, 2023), machine learning (ML) and item response theory (IRT) analytics may be particularly useful for analyzing large volumes of interrelated, nonlinear, and high-dimensional data at the item level (Reise and Waller, 2009; Mohri et al., 2012).

Not only can these approaches analyze more complex data but they can also improve the development and refinement of PVTs relative to classical measurement approaches. For example, person-fit statistics is an IRT approach that has been used to identify non-credible symptom reporting in dichotomous and polytomous data (Beck et al., 2019). This approach may also improve embedded PVTs by estimating the extent to which each item-level response deviates from one's true abilities (Bilder and Reise, 2019). Scott et al. (2023) found that using person-fit statistics helped embedded PVTs detect subtle patterns of non-credible performance. IRT is especially amenable to computerized adaptive testing, which adjusts each item's difficulty based on one's response. Computerized adaptive testing systems can create shorter and more precise PVTs with psychometrically equivalent alternative forms (Gibbons et al., 2008). These systems can also detect careless responding based on unpredictable error patterns that deviate from normal difficulty curves. Detecting careless responding may be useful for PVTs embedded within digital self-paced continuous performance tests (e.g., Nicholls et al., 2020; Berger et al., 2021). Other IRT approaches can improve PVTs by scrutinizing item difficulty and discriminatory power and identifying culturally biased items. For example, differential item functioning is an IRT approach that may identify items on English-verbally mediated PVTs that are disproportionately challenging for those who do not speak English as their primary language, allowing for appropriate adjustments.

ML has proven useful in symptom validity test development (Orrù et al., 2021) and may function similarly for PVTs. Two studies recently investigated whether supervised ML improves PVA (Pace et al., 2019; Hirsch et al., 2022). Pace et al. (2019) found that a supervised ML model trained with various features (demographics, cognitive performance errors, response time, and a PVT score) discriminated between genuine and simulated cognitive impairment with high accuracy. Using similar features, Hirsch et al. (2022) found that their supervised models had moderate to weak prediction of PVT failure in a clinical attention-deficit/hyperactivity disorder sample. No studies have used unsupervised ML for PVA. It is possible that unsupervised ML could also identify groups of credible and non-credible performing examinees using relevant factors such as PVT scores, litigation status, medical history, and referral reasons, without explicit programming. Software can be developed to extract data for the ML via computerized questionnaires or electronic medical records. Deep learning, a form of ML that processes data using multiple dimensions, may also detect complex and anomalous patterns indicative of non-credible performance. Deep learning may be especially useful for analyzing response sequences over time (e.g., non-credible changes in performance across repeat medico-legal evaluations). Furthermore, deep-learning models may be effective at identifying inherent statistical dependencies and patterns of non-credible performance, and thus generating expectations of how genuine responses should appear. Combining these algorithms with other statistical techniques that assess response complexity and highly anomalous responses (e.g., Lundberg and Lee, 2017; Parente and Finley, 2018; Finley and Parente, 2020; Orrù et al., 2020; Mertler et al., 2021; Parente et al., 2021, 2023; Finley et al., 2022; Rodriguez et al., 2024), may increase the signal of non-credible performance. These algorithmic approaches can improve as we better understand cognitive phenotypes and what is improbable for certain disorders using precision medicine and bioinformatics.

## Facilitating scale and sustainability

To optimize the utility of these digital data, technologies can include point-of-testing acquisition software that automatically transfers data to cloud-based, centralized repositories. These repositories facilitate sustainable and scalable innovations by increasing data access and collaboration among PVA stakeholders (see Reeves et al., 2007 and Gaudet and Weyandt, 2017 for large-scale developments of digital tests with embedded PVTs). Multidisciplinary approaches are needed to make theoretical and empirical sense of the data collected via digital technologies (Collins and Riley, 2016). With more comprehensive and uniform data amenable to data mining and deep-learning analytics, collaborating researchers can address overarching issues that remain poorly understood within research. For example, with larger centralized data researchers can directly evaluate different statistical approaches (e.g., chaining likelihood ratios vs. multivariable discriminant function analysis, Bayesian model averaging, or logistic regression) as well as the joint validity of standardized test batteries (Davis, 2021; Erdodi, 2023; Jewsbury, 2023). Such data and findings could also help determine robust criterion-grouping combinations, given that multiple PVTs assessing complementary aspects of performance across various cognitive domains may be necessary for a strong criterion-grouping combination (Schroeder et al., 2019; Soble et al., 2020). Similarly, researchers could expand upon existing decision-making models (e.g., Rickards et al., 2018; Sherman et al., 2020) by using these comprehensive data to develop algorithms that automatically generate credible/non-credible profiles based on the type and proportion or number of PVTs failed in relation to various contextual and diagnostic factors, symptom presentations, and clinical inconsistencies (across medical records, self- and informant-reports, or behavioral observations). A greater range and depth of data may further help elucidate the extent to which several putative factors—such as bona fide injury/disease, normal fluctuation and variability in testing, level of effort (either to perform well or to deceive), and symptom validity, among others—are associated with performance validity (Larrabee, 2012; Bigler, 2014). Understanding these associations could help identify the mechanisms underlying non-credible performance.

Collaboration is especially needed for basic and applied sciences to coalesce unique aspects of PVA that have been studied independently, such as integrating neuropsychology and neurocognitive processing theories to develop more sophisticated stimuli/paradigms (Leighton et al., 2014). For example, less applied scientific models, such as memory familiarity vs. conscious recollection theories, may be applied to clinically available PVTs to reduce false-positive rates in certain neurological populations (Eglit et al., 2017). Similar areas of cognitive science have also shown that using pictorial or numerical stimuli (vs. words) across multiple learning trials can reduce false-positive errors in clinical settings (Leighton et al., 2014). Furthermore, integrating data in real time into these repositories offers a sustainable and accurate way of estimating PVT failure base rates and developing cutoffs accordingly. Finally, as proposed by the National Neuropsychology Network (Loring et al., 2022), a centralized repository for digital data that is backward-compatible with analog test data can provide a smooth transition from traditional pencil-and-paper tests to digital formats. These repositories (including those curated via the National Neuropsychology Network) thus enable sustainable innovation by supporting continuous incremental refinement of PVTs over time.

## Increasing accessibility

As observed in other areas of neuropsychology (Miller and Barr, 2017), digital technologies can offer more accessible PVA. Specifically, web-based PVTs can help access underserved and geographically restricted communities, but with the understanding that disparities in digital technology may also exist. Although more web-based PVTs are needed, not every PVT requires digitization for telehealth (e.g., Reliable Digit Span; Kanser et al., 2021; Harrison and Davin, 2023). Digital PVTs can also increase accessibility in primary care settings where digital cognitive screeners are being developed for face-to-face evaluations and may be completed in distracting, unsupervised environments (Zygouris and Tsolaki, 2015). Validity indicators could be embedded within these screeners rather than creating new freestanding PVTs. The National Institutes of Health Toolbox^®^ (Abeare et al., 2021) and Penn Computerized Neurocognitive Battery (Scott et al., 2023) are well-established digital screeners with embedded PVTs that offer great promise for these evaluations. In primary care, embedded PVTs could serve as preliminary screeners for atypical performance that warrants further investigation. Digital PVTs may also increase accessibility in research settings. Although it is not highly likely research volunteers would deliberately feign impairment, they may lose interest, doze off, or rush through testing (An et al., 2017), especially in dementia-focused research where digital testing is common. Some digitally embedded PVTs have been developed for ADHD research (Table 1) and may be used in other research focused on digital cognitive testing (Bauer et al., 2012).

## Enhancing efficiencies

Finally, the application of digital technologies introduces new efficiencies; in PVA, they hold the promise of improved standardization and administration/scoring accuracy. Technologies can leverage automated algorithms to reduce time spent on scoring and routine aspects of PVA (e.g., finding/adjusting PVT cutoffs according to various contextual/intrapersonal factors). Automation would allow providers to allocate more time to case conceptualization and responding to (rather than detecting) validity issues. Greater efficiencies in PVA translate into greater cost-efficiencies as well as reduced collateral expenses for specialized training, testing support, and materials (Davis, 2023). Further, digital PVTs can automatically store, retrieve, and analyze data to generate multiple relevant scores (e.g., specificity, sensitivity, predictive power adjusted for diagnostic-specific base rates, false-positive estimates, and likelihood ratios or probability estimates for single/multivariable failure combinations). Automated scoring will likely become increasingly useful as more PVTs and data are generated.

## Limitations and concluding remarks

By no means an exhaustive review, this paper describes five ways in which digital technologies can improve PVA. These improvements can complement rather than replace the uniquely human aspects of PVA. Thus, the upfront investments required to transition to digital approaches are likely justifiable. However, other limitations deserve attention before making this transition. As described elsewhere (Miller and Barr, 2017; Germine et al., 2019), limitations to digital assessment may include variability across devices, which can impose different perceptual, motor, and cognitive demands that affect the reliability and accuracy of the tests. Variations in hardware and software within the same class of devices can affect stimulus presentation and response (including response latency) measurement. Individual differences in access to and familiarity with technology may further affect test performance. Additionally, the rapid advancement in technologies suggests that hardware and software can quickly become obsolete. A large influx of data and the application of “black box” ML algorithms and cloud-based repositories also raises concerns regarding data security and privacy. Addressing these issues and implementing digital methods into practice or research would require substantial technological and human infrastructure that may not be attainable in certain settings (Miller, 2019). Indeed, the utility of digital assessments likely depends on the context in which they are implemented. For example, PVA is critical in forensic evaluations but the limitations described above could challenge compliance with the evolving standards for the admissibility of scientific evidence in these evaluations. Further discussion of these limitations along with the logistical and practical considerations for a digital transition is needed (for further discussion, see Miller, 2019; Singh and Germine, 2021). Finally, other digital opportunities, such as using validity indicators with ecological momentary assessment and virtual reality technologies, merit further discussion. Moving forward, scientists are encouraged to expand upon these digital innovations to ensure that PVA evolves alongside the broader landscape of digital neuropsychology.

## Author contributions

J-CF: Conceptualization, Investigation, Methodology, Resources, Supervision, Writing – original draft, Writing – review & editing.
